# The LDL1/2-HDA6 Histone Modification Complex Interacts With TOC1 and Regulates the Core Circadian Clock Components in *Arabidopsis*

**DOI:** 10.3389/fpls.2019.00233

**Published:** 2019-02-26

**Authors:** Fu-Yu Hung, Fang-Fang Chen, Chenlong Li, Chen Chen, Jian-Hao Chen, Yuhai Cui, Keqiang Wu

**Affiliations:** ^1^Institute of Plant Biology, National Taiwan University, Taipei, Taiwan; ^2^Agriculture and Agri-Food Canada, London Research and Development Centre, London, ON, Canada; ^3^Department of Biology, Western University, London, ON, Canada; ^4^State Key Laboratory of Biocontrol and Guangdong Key Laboratory of Plant Resource, School of Life Sciences, Sun Yat-sen University, Guangzhou, China

**Keywords:** H3K4 demethylases, HDA6, circadian clock, CCA1/LHY, TOC1, *Arabidopsis*

## Abstract

In *Arabidopsis*, the circadian rhythm is associated with multiple important biological processes and maintained by multiple interconnected loops that generate robust rhythms. The circadian clock central loop is a negative feedback loop composed of the core circadian clock components. *TOC1* (*TIMING OF CAB EXPRESSION 1*) is highly expressed in the evening and negatively regulates the expression of *CCA1* (*CIRCADIAN CLOCK ASSOCIATED 1*)/*LHY* (*LATE ELONGATED HYPOCOTYL*). CCA1/LHY also binds to the promoter of *TOC1* and represses the *TOC1* expression. Our recent research revealed that the histone modification complex comprising of LYSINE-SPECIFIC DEMETHYLASE 1 (LSD1)-LIKE 1/2 (LDL1/2) and HISTONE DEACETYLASE 6 (HDA6) can be recruited by CCA1/LHY to repress *TOC1* expression. In this study, we found that HDA6, LDL1, and LDL2 can interact with TOC1, and the LDL1/2-HDA6 complex is associate with TOC1 to repress the *CCA1/LHY* expression. Furthermore, LDL1/2-HDA6 and TOC1 co-target a subset of genes involved in the circadian rhythm. Collectively, our results indicate that the LDL1/2-HDA6 histone modification complex is important for the regulation of the core circadian clock components.

## Introduction

The circadian rhythm is an endogenous oscillation widely observed in plants, animals, fungi, and cyanobacteria (Edgar et al., 2012). The plant circadian rhythm is highly associated with multiple important biological processes, and maintained by multiple interconnected loops that generate robust rhythms. The circadian clock central loop is a negative feedback loop composed of the core circadian clock components such as *TOC1* (*TIMING OF CAB EXPRESSION 1*) and *CCA1* (*CIRCADIAN CLOCK ASSOCIATED 1*)/*LHY* (*LATE ELONGATED HYPOCOTYL*). *TOC1* is highly expressed in the evening, but low expressed at dawn (Alabadi et al., 2001). Furthermore, TOC1 was identified as a repressor of *CCA1* and *LHY* by binding to their promoters in the evening (Gendron et al., 2012; Huang et al., 2012). In contrast, *CCA1* and *LHY* are highly expressed in the morning, but low expressed at nightfall (Schaffer et al., 1998; Wang and Tobin, 1998; Alabadi et al., 2001). CCA1 and LHY bind to the evening element (EE) on the promoter of *TOC1* to inhibit its expression (Schaffer et al., 1998; Wang and Tobin, 1998; Alabadi et al., 2001; Nagel et al., 2015). CHE (CCA1 HIKING EXPEDITION) is an evening-expressed TCP-family transcription factor, which also targets the *CCA1* promoter to repress its expression. Furthermore, CCA1 and LHY were shown to repress the *CHE* expression by targeting the *CHE* promoter (Pruneda-Paz et al., 2009).

Histone modifications play important roles in the regulation of gene expression. Histone methyltransferases and demethylases determine the methylation levels, whereas histone acetylation levels are regulated by histone acetyltransferases (HATs) and histone deacetylases (HDACs or HDAs). HDACs and the H3K4 demethylase LSD1 (Lysine-Specific Demethylase 1) are the core components of the Mi2/NuRD and CoREST protein complexes in yeast and animal cells (Khochbin et al., 2001; Lee et al., 2005; Wang et al., 2009). They act co-operatively to repress gene expression in mammals (Huang et al., 2011). The interactions among the core protein components of the HDAC complexes are relatively stable and the HDAC complexes can also interact with various transcription factors under different environmental conditions (Joshi et al., 2013; Liu et al., 2014). FLD (FLOWERING LOCUS D), LDL1 (Lysine-Specific Demethylase-LIKE 1), LDL2, and LDL3 are the LSD1 homologs in *Arabidopsis* (Jiang et al., 2007). LDL1 and LDL2 act redundantly to regulate *FLC (FLOWERING LOCUS C)* by H3K4 demethylation (Jiang et al., 2007). Furthermore, *Arabidopsis* HISTONE DEACETYLASE 6 (HDA6) directly interacts with FLD to repress *FLC*, *MAF4*, and *MAF5* by reducing H3K4 methylation (H3K4me) and H3 acetylation (H3Ac) to regulate flowering time (Yu et al., 2011). In addition, HDA6 can also interact with LDL1 and LDL2 to regulate gene expression (Hung et al., 2018).

The HDAC inhibitor TSA treated plants show delayed phases and higher amplitudes of *TOC1* expression (Perales and Más, 2007). In addition, the expression of *Arabidopsis CCA1*, *LHY*, and *TOC1* is specifically associated with H3Ac and H3K4me changes (Hemmes et al., 2012; Malapeira et al., 2012), indicating that the expression of the core circadian clock components is associated with H3Ac and H3K4me level changes. Our recent study indicated that CCA1 and LHY can interact with the HDAC complex containing LDL1, LDL2, and HDA6. Furthermore, the LDL1/2-HDA6 complex can be recruit by the transcription repressors CCA1 and LHY to their target genes including *TOC1*. Since *CCA1* and *LHY* are low expressed at nightfall, the expression of *TOC1* is increased due to the release of LDL1/2-HDA6 from the *TOC1* promoter (Hung et al., 2018). In this study, we demonstrated that LDL1/2-HDA6 can also interact with TOC1 to regulate the expression of *CCA1* and *LHY*. Furthermore, LDL1/2-HDA6 and TOC1 co-target a subset of genes involved in the circadian rhythm.

## Materials and Methods

### Plant Materials and Growth Conditions

The *Arabidopsis thaliana* Columbia (Col-0) ecotype was used. Plants were grown at 22°C under 12/12 h light/dark conditions in growth chambers. The mutants used in this study were previously described, including *ldl1/ldl2* (Jiang et al., 2007), *hda6* (*axe1-5*) (Yu et al., 2011), *hda6/ldl1/2* (Hung et al., 2018), *toc1*, and *cca1/lhy* (Wang et al., 2011). *35Spro::LDL1:GFP*, *35Spro::GFP:HDA6*, *LDL1pro::LDL1:GFP* and *HDA6pro::HDA6:GFP* transgenic plants were previously described (Yu et al., 2011; Hung et al., 2018).

The full-length coding sequence (CDS) fragment of *TOC1* was PCR-amplified and cloned into the *pCR8/GW/TOPO* vector (Invitrogen), and then recombined into the *PK7WGF2* binary vector or *3xFLAG* Gateway vector (Invitrogen^1^). The *35S::TOC1:GFP* vector was transformed into Col-0 WT or *hda6/ldl1/2* by the floral dip method.

### Bimolecular Fluorescence Complementation (BIFC) Assays

To generate the constructs for BiFC assays, the full-length coding sequence (CDS) fragment of *TOC1* was amplified by PCR and cloned into the *pCR8/GW/TOPO* vector, and then recombined into the *pEarleyGate201-YN* (Lu et al., 2010). *LDL1-YC* and *HDA6-YC* were described in the previous studies (Yu et al., 2011; Hung et al., 2018). Constructed vectors were transformed into *Arabidopsis* protoplasts or tobacco (*Nicotiana benthamiana*) leaves for transient assays. Transformed protoplasts and tobacco leaves were then examined by confocal spectral microscope imaging system (NTU-TCS SP5, Leica^2^).

### Yeast Two-Hybrid (Y2H) Assays and Co-immunoprecipitation (Co-IP) Assays

Yeast two-hybrid assays were performed based on the instruction for the Matchmaker GAL4-based two-hybrid system 3 (Clontech). The *LDL1*, *LDL2*, and *TOC1* full length cDNA fragments were sub-cloned into *pGADT7* and *pGBKT7* vectors. All constructs were transformed into the yeast (*Saccharomyces cerevisiae*) strain AH109 by the lithium acetate method, and yeast cells were grown on a minimal medium/-Leu-Trp according to the manufacturer’s instructions (Clontech). Transformed colonies were grown on the medium containing X-α-gal for the α-galactosidase activity assay or minimal medium/-Leu-Trp-His (3DO) with 0.25 mM 3-amino- 1,2,4-triazole (3AT).

Co-immunoprecipitation assays were performed as previously described (Yu et al., 2011). The *35S::TOC1:3xFLAG* plasmid was transformed into *Arabidopsis* protoplasts extracted from *LDL1pro::LDL1:GFP* or *35Spro::GFP* transgenic plants. Total proteins were than extracted from the transformed protoplasts. Anti-GFP (Santa Cruz Biotechnologies, catalog no. SC-9996; 1:3000 dilution) and anti-FLAG (SIGMA catalog no. M2; 1:3000 dilution) antibodies were used as primary antibodies for Western blot. The resulting signals were detected by using a Pierce ECL Western blotting kit (Pierce^3^).

### Quantitative Real-Time PCR (qRT-PCR) Analysis

The TRIZOL reagent (Invitrogen, 15596026) was used for total RNA isolation according to the manufacturer’s instructions. Total RNA treated with 2 μg of DNAse (Promega, RQ1 #M6101) were then used for cDNA synthesis (Promega, #1012891). The iQ SYBR Green Supermix solution (Bio-Rad, #170-8880) was used for real-Time quantitative PCR assays with the CFX96 real-time PCR Detection System (Bio-Rad Laboratories, Inc.). Cycling conditions were started with 95°C/10 min, followed by 45 cycles of 95°C/15 s, 60°C/30 s, and then fluorescent detection, and melting curve detection (65–95°C, incrementing 0.5°C for 5 s, and plate reading). Each sample was normalized by calculating delta quantification cycle (Cq) to the expression of the *UBQ10* (*Ubiquitin10*) internal control and quantified at least in triplicate. The Cq and relative expression level are calculated by the Biorad CFX Manager 3.1 based on the MIQE guidelines (Bustin et al., 2009). Supplementary Table S1 listed the gene specific primers used for qRT-PCR. Standard deviations (SD) represent at least three technical and three biological replicates. The variance in average data is represented by standard error of the mean (SEM). The SD, SEM determination and *P*-value were calculated using Student’s paired *t*-test.

### Protoplast Transient Assays

The *CCA1pro::LUC* plasmid construct was previously described (Wang et al., 2011). For transcriptional activity assays, the *35Spro::TOC1*, *35Spro::LDL1*, *35Spro::HDA6*, or *35Spro::GFP* effector constructs were co-transformed into protoplasts with *CCA1pro::LUC*, and the plant samples were collected at ZT0 after 12 h. The relative activities of LUC (luciferase) reporter were standardized by activities of co-expressed Renilla LUC. Experiments were repeated at least three times for each reporter-effector combination. The dual luciferase assay reagent (Promega) was used for Firefly LUC and Renilla LUC detection.

### Chromatin Immunoprecipitation (ChIP) Assays and ChIP-seq Data Analyses

Chromatin immunoprecipitation assays were accomplished as previously described (Yu et al., 2011; Hung et al., 2018). Plant seedlings were treated with 1% formaldehyde for chromatin extraction. The extracted DNA was sheared to the mean length near 500 bp by sonication, proteins, and DNA fragments were then immunoprecipitated by the H3K9K14 (Millipore, catalog no. 06-599), H3K4me3 (Milipore, catalog no. 04-745), or GFP (Abcam, catalog no. ab290) antibodies. The cross-link between DNA with immunoprecipitated proteins were reversed, and then analyzed by real-time PCR using specific primers (Supplementary Table S1). The quantification cycle(Cq) was calculated by Biorad CFX Manager 3.1 based on the MIQE guideline (Bustin et al., 2009). Percent input was calculated as 2^∧^[Cq(IN)-Cq(IP)]X100. Each sample was quantified at least in triplicate, and normalized by calculating delta Cq to the expression of the internal control. Standard deviations (SD) represent at least three technical and three biological replicates. The variance in average data is represented by standard error of the mean (SEM). The SD, SEM determination and *P*-value were calculated using Student’s paired *t*-test.

ChIP-seq assays were performed based on previous research (Li et al., 2015, 2016; Hung et al., 2018). The LDL1 ChIP-seq data were deposited to NCBI-Gene Expression Omnibus (GEO) database (GSE118025) (Hung et al., 2018). The ChIP-Seq files from other research groups, GSE35952 (Huang et al., 2012) and (Kamioka et al., 2016), were downloaded from the NCBI-GEO database.

## Results

### LDL1 and HDA6 Interact With TOC1 and Directly Target on *CCA1* and *LHY*

**Figure 1:**
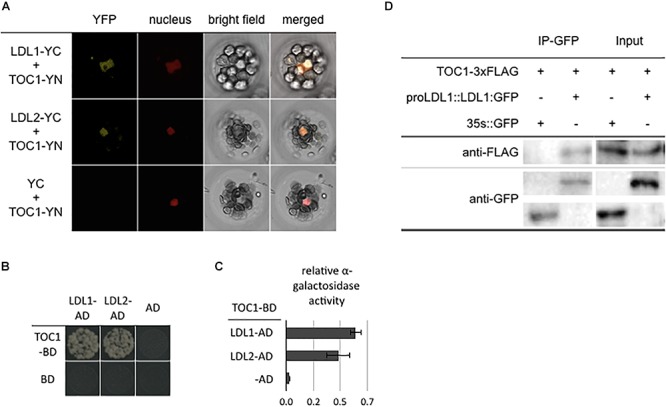
LDL1/LDL2 interact with TOC1. **(A)** BiFC assays in *Arabidopsis* protpplasts showing interaction between LDL1/LDL2 and TOC1 in living cells. LDL1, LDL2, and TOC1 fused with the N terminus (YN) or C terminus (YC) of YFP were co-delivered into *Arabidopsis* protpplasts. The nucleus was indicated by mCherry carrying a nuclear localization signal. **(B,C)** Yeast two hybrid analysis of the interaction of LDL1/LDL2 with TOC1. LDL1-BD/LDL2-BD with CCA1-AD or LHY-AD was co-transformed into the yeast strain AH109. The transformants were plated on the SD/-Leu-Trp-His medium. **(C)** Quantitative α-galactosidase assays for protein-protein interaction in yeast. Bars indicate SD from three biological replicates. **(D)** Co-IP of the native promoter driven LDL1:GFP with TOC1 in *LDL1pro::LDL1:GFP* transformed *Arabidopsis* protoplasts. Western blot (WB) was performed with the anti-FLAG and anti-GFP antibodies.

Our recent study indicated that CCA1/LHY can interact with the LDL1/2-HDA6 complex to repress *TOC1* (Hung et al., 2018). In addition, the expression of *TOC1*, *CCA1* and *LHY* is also associated with H3K4me and H3 acetylation changes (Hemmes et al., 2012; Malapeira et al., 2012). We further analyzed the functional correlation between TOC1 and the LDL1/2-HDA6 complex. TOC1 directly interacted with both LDL1 and LDL2 in BiFC assays by using *Arabidopsis* protoplasts and *Agrobacterium*-infiltrated tobacco leaves. The YFP fluorescence signal was detected in nucleus of the transformed cells (Figure 1A and Supplementary Figure S1). The interaction between LDL1, LDL2, and TOC1 was further confirmed by yeast two-hybrid assays (Figure 1B,C) and Co-IP assays using *Arabidopsis* protoplasts (Figure 1D and Supplementary Figure S1). Furthermore, TOC1 can also interact with HDA6 in BiFC assays (Supplementary Figures S2A,B). These results suggested that TOC1 may recruit the LDL1/2-HDA6 histone modification complex to its target genes such as *CCA1* and *LHY*.

**Figure 2:**
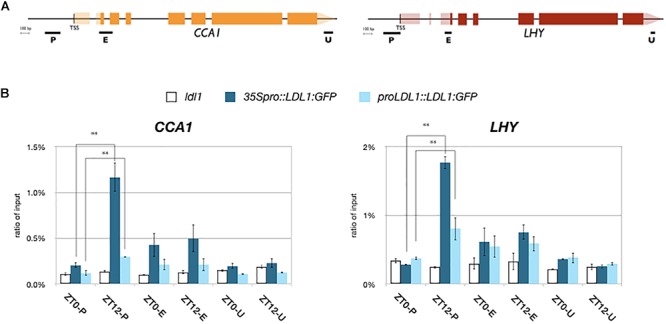
LDL1/LDL2 target on *CCA1* and *LHY*. **(A)** Schematic diagram of *CCA1* and *LHY*. P: promoter region, E: coding region, U: 3′ UTR. **(B)** LDL1 bindis to the *CCA1* and *LHY* promoters. *35S pro::LDL1:GFP* or *LDL1pro::LDL1:GFP* was transformed into *ldl1*. 14 days-old seedlings grown under 12/12: light/dark were harvested on ZT0 or ZT12. ChIP assays were performed with the anti-GFP antibody. The amount of immunoprecipitated DNA was quantified by qRT-PCR. Values represent the average immunoprecipitation efficiencies (%) against the total input DNA. Error bars correspond to standard deviations from three biological replicates. ^∗^*P* < 0.05, ^∗∗^*P* < 0.005 (Student’s *t*-test).

We further analyzed the binding of LDL1 and HDA6 to *CCA1* and *LHY* by ChIP assays. The *LDL1:GFP* and *HDA6:GFP* transgenic plants were previously described (Yu et al., 2011; Hung et al., 2018). 14 days old plants grown under 12 h light/12 h dark condition were collected on Zeitgeber time 0 (ZT0) and ZT12. An anti-GFP antibody was used for ChIP assays, and the binding of LDL1 and HDA6 was analyzed by qPCR. We identified that both LDL1 and HDA6 can bind to the promoters of *CCA1* and *LHY*. Furthermore, the binding of LDL1 and HDA6 to the promoters of *CCA1* and *LHY* were significantly decreased on ZT0 compared to ZT12 (Figure 2 and Supplementary Figure S2C). The binding of LDL1 and HDA6 to the *CCA1* and *LHY* promoters is correlated to TOC1 accumulation, since *TOC1* is highly expressed at nightfall but low expressed in the morning (Alabadi et al., 2001).

### TOC1 and LDL1 Co-target Genes Involved in the Circadian Rhythm

Previously, we identified the global binding sites of LDL1 by ChIP-Seq assays (Hung et al., 2018). The GO-BP (Gene Ontology_Biological Process) analysis of LDL1-targeted genes revealed that LDL1 targets on a subset of circadian rhythm genes. Furthermore, LDL1 also binds to a cluster of circadian rhythm genes regulated by CCA1 (Hung et al., 2018). In this study, we further analyzed whether the LDL1 and TOC1 also co-target genes involved in the circadian rhythm.

**Figure 3:**
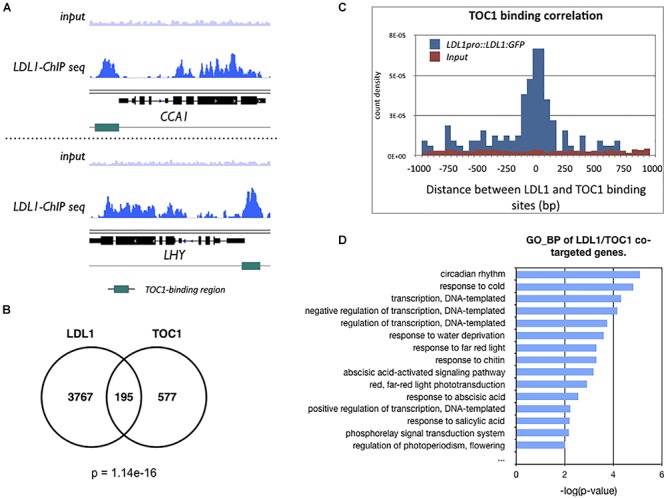
LDL1-occupied sites in the genome identified by ChIP-seq analysis. **(A)** Integrated genome view of LDL1 binding peaks on *CCA1* and *LHY*. green BARS indicate the TOC1-binding regions form previous published data (Huang et al., 2012). **(B)** Overlap between TOC1 target genes (Huang et al., 2012) and LDL1 targeted genes (Hung et al., 2018) (hypergeometric distribution of TOC1 and LDL1 co-targeted genes: *p* = 1.14e–16). **(C)** Distribution of distances between the total binding sites of LDL1 and TOC1. **(D)** GO-BP annotation of LDL1/TOC1 co-occupied genes. Annotation terms with *p*-value < 0.01 were listed.

We compared the previously published TOC1 ChIP-Seq data (Huang et al., 2012) with the LDL1 ChIP-Seq data (Hung et al., 2018). The genome browser views by Integrative Genomics Viewer (IGV) indicated that LDL1 bound to *CCA1* and *LHY*, and the binding peaks of LDL1 are highly correlated with the TOC1 binding regions on *CCA1* and *LHY* promoters (Figure 3A). Among 772 genes occupied by TOC1 (Huang et al., 2012), 195 of them are also co-occupied by LDL1 (*P* = 1.14e-16) (Figure 3B). Furthermore, the genomic binding regions of TOC1 are closed to the LDL1 binding regions (Figure 3C), indicating that TOC1 and LDL1 tend to bind to the similar genome sites. GO-BP analysis also indicated that LDL1 and TOC1 co-target on a subgroup of genes involved in circadian rhythm and response to cold (Figure 3D). In GO-BP analysis, the ratio of the circadian genes of LDL1/TOC1 co-targeted genes is increased when compared to the LDL1-targeted genes or the TOC1-targeted genes alone (Supplementary Figure S3). Interestingly, the ratio of the circadian rhythm genes is further increased in the LDL1/CCA1/TOC1 co-targeted genes (Supplementary Figure S3). Previous studies indicated that several *cis*-elements are enriched in the promoters of TOC1 regulated genes, including the (AG/CT)_n_ repeat, G-box (CACGTG), Evening Element (EE)-like and TCP binding site (TBS, GGCCCA) (Gendron et al., 2012; Huang et al., 2012). Similar *cis*-elements are also enriched in the LDL1-targeted promoter regions (Hung et al., 2018).

### LDL1/2-HDA6 Is Involved in the Regulation of *CCA1/LHY*

**Figure 4:**
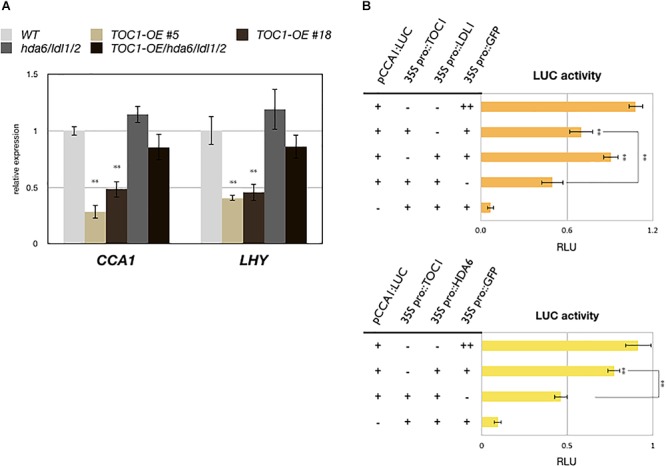
LDL1/2-HDA6 is involved in regulation of *CCA1/LHY*. **(A)** Expression of CCA1 and LHY in *TOC1-OE* plants, *hda6/ldl1/2*, and WT. Gene expression levels were determined by qRT-PCR and normalized to *UBQ10*. Plants were grown under 12/12 light/dark for 14 days and collected on ZT0. **(B)** Transient luciferase assays in *CCA1pro::CCA1:LUC* (*pCCA1:LUC*) transformed protoplasts. CaMV 35S promoter driven *TOC1*, *HDA6*, or *LDL1* effector constructs were introduced into mesophyll protoplasts. Samples were collected on ZT0 after 12 h of transformation. Relative Light Units (RLU) represents firefly luciferase normalized by co-expressed *35S pro::Renilla luciferase*. *35Spro::GFP* transformed protoplasts were used as the negative control. Data points represent the average of three technical replicates. Error bars correspond to SD from three biological replicates. ^∗^*P* < 0.05, ^∗∗^*P* < 0.005 (Student’s *t*-test).

TOC1 is a repressor and targets on the promoters of *CCA1* and *LHY*. The expression of *CCA1* and *LHY* is decreased in *TOC1* over-expressing (*TOC1-OE*) plants (Gendron et al., 2012; Huang et al., 2012). Furthermore, additional *TOC1* expression causes increased period length of *CCA1* (Mas et al., 2003a). To investigate the functional relationship between TOC1 and LDL1/2-HDA6, we generated *TOC1 over-expressing* plants in WT (*TOC1-OE*) and the *hda6/ldl1/2* background (*TOC1-OE/hda6/ldl1/2*). The binary vector containing *CaMV 35S promoter* driven *GFP:TOC1* (*35S::GFP:TOC1*) was transformed into WT or *hda6/ldl1/2*. The expression patterns of *CCA1* and *LHY* were compared by qRT-PCR in wild-type (WT), *TOC1-OE* and *ldl1/2/hda6* plants grown under 12 h light/12 h dark for 14 days. As reported previously (Gendron et al., 2012; Huang et al., 2012), the expression of *CCA1* and *LHY* was decreased in *TOC1-OE* plants. However, the expression of *CCA1* and *LHY* was not significantly decreased in *hda6/ldl1/2* compared to WT (Figure 4A and Supplementary Figures S4A,B). Furthermore, the decrease of *CCA1* and *LHY* expression was recovered when *TOC1* was over-expressed in *hda6/ldl1/2* (Figure 4A and Supplementary Figures S4B,C). We also compared the daily expression patterns of *CCA1*, *LHY*, and *TOC1* in *ldl1/ldl2*, *hda6*, *hda6/ldl1/2*, and WT grown under 12 h light/12 h dark conditions. The expression of *CCA1* and *LHY* was not significantly decreased or shifted in *ldl1/ldl2*, *hda6*, and *hda6/ldl1/2* compared to WT (Supplementary Figure S4A). The expression patterns of other TOC1 targets such as *GI*, *PRR7* and *PRR9* in *ldl1/ldl2*, *hda6*, and *hda6/ldl1/2* were analyzed in our previous study (Hung et al., 2018). *XTH27* and *AT1G10020* were previously identified to be the target genes regulated by TOC1 (Gendron et al., 2012; Huang et al., 2012), which are also targeted by LDL1 (Hung et al., 2018). The expression of *XTH27* and *AT1G10020* was increased in *ldl1/ldl2*, *hda6*, and *hda6/ldl1/2* compared to WT (Supplementary Figure S4C).

We further analyzed the functional correlation between LDL1, HDA6, and TOC1. *CCA1pro::CCA1:LUC* (*pCCA1:LUC*) was co-expressed with *35Spro::TOC1*, *35Spro::LDL1*, *35Spro::HDA6*, or *35Spro::GFP* in *Arabidopsis* protoplasts. Although the activity of *CCA1:LUC* was only slightly reduced when co-expressed with LDL1, and activity was further decreased when TOC1 was co-expressed with LDL1 (Figure 4B). Similar results were also observed when TOC1 was co-expressed with HDA6 (Figure 4C).

**Figure 5:**
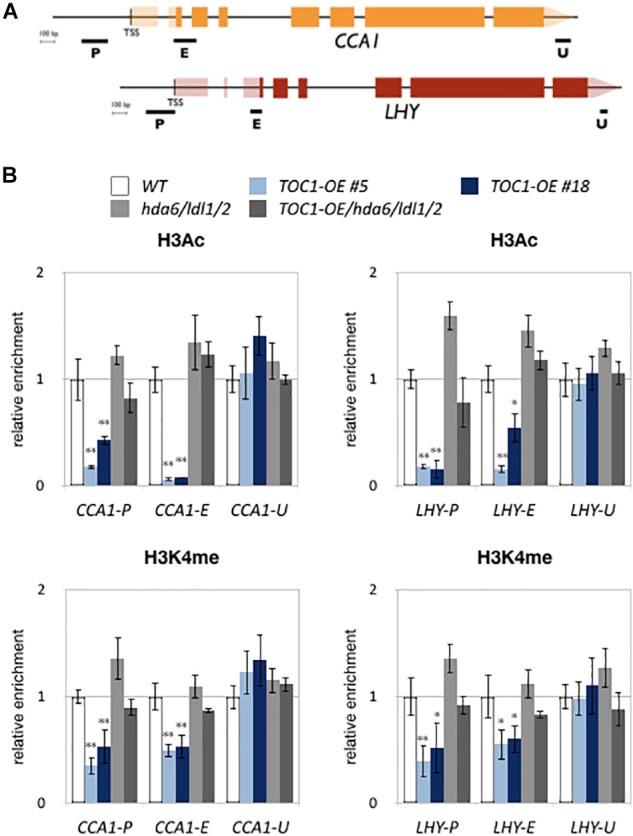
LDL1/2-HDA6 is involved in regulation of H3Ac and H3K4me of *CCA1/LHY*. (A) Schematic diagram of CCA1 and LHY. P: promoter region, E: coding region, U: 3′ UTR. (B) ChIP analysis of H3ac and H3K4me levels of *CCA1* and *LHY* in *TOC1-OE* plants on ZT0. The amounts of DNA after ChIP were quantified by qRT-PCR and normalized to *ACT2*. Plants were grown under 12/12 : light/dark for 14 days. Data points represent the average of three technical replicates. Error bars correspond to SD from three biological replicates. ^∗^*P* < 0.05, ^∗∗^*P* < 0.005 (Student’s *t*-test).

We also analyzed H3K4me and H3Ac levels of *CCA1* and *LHY* in WT, *TOC1-OE* plants and *hda6/ldl1/2*. For ChIP-qPCR assays, 14-days old plants grown under 12 h light/12 h dark conditions were collected on ZT0. H3K4me and H3Ac of *CCA1* and *LHY* were decreased in *TOC1-OE* plants (Figure 5), indicating that TOC1 affects the levels of H3K4me and H3Ac on *CCA1* and *LHY*. We further analyzed H3Ac and H3K4me levels of *CCA1* and *LHY* in 14 days old *hda6*, *ldl1/ldl2*, *hda6/ldl1/2*, and WT on ZT0 and ZT12. The H3Ac and H3K4me levels of *CCA1* and *LHY* were not decreased in *hda6*, *ldl1/ldl2*, *hda6/ldl1/2* (Supplementary Figure S4D). Interestingly, decreased H3K4me and H3Ac in *TOC1-OE* were recovered in *TOC1-OE/hda6/ldl1/2*, since the H3Ac and H3K4me levels of *CCA1* and *LHY* were significant higher in *TOC1-OE/hda6/ldl1/2* compared to the *TOC1-OE* plants (Figure 5). These results suggested that TOC1 is involved in regulation of H3K4me and H3Ac on *CCA1* and *LHY*, and TOC1 repressed *CCA1* and *LHY* expression is dependent on the function of LDL1/2-HDA6 complex.

## Discussion

*Arabidopsis* HDA6 is a class I RPD3-like histone deacetylase associated with regulation of rRNA and transcription repression (Murfett et al., 2001; Probst et al., 2004; Earley et al., 2006; Liu et al., 2012; Yu et al., 2017). Different transcription factors can recruit HDA6 to regulate the gene expression involved in flowering, leaf development, abiotic stress response, and senescence (Wu et al., 2008; Chen et al., 2010; Yu et al., 2011; Luo et al., 2012; Liu et al., 2014). In animal and yeast cells, HDACs and LSD1 regulate gene expression cooperatively and they are both identified as the core components of Mi2/NuRD and CoREST complexes (Khochbin et al., 2001; Lee et al., 2005; Wang et al., 2009). Our recent study demonstrated that the *Arabidopsis* H3K4 demethylases LDL1 and LDL2 can interact with HDA6 to repress gene expression (Hung et al., 2018). The LDL1/2-HDA6 complex can also interact with CCA1/LHY and reduce H3Ac and H3K4me levels of the circadian core component *TOC1* (Hung et al., 2018). Furthermore, a subset of genes involved in the circadian clock are co-targeted by LDL1 and CCA1 (Hung et al., 2018).

*Arabidopsis* circadian clock genes are regulated by a complicate feedback regulation network forming multiple interconnected loops. The central loop is comprised of the core clock components, such as TOC1 and CCA1/LHY (Schaffer et al., 1998; Wang and Tobin, 1998; Alabadi et al., 2001; Gendron et al., 2012; Huang et al., 2012; Nagel et al., 2015). The central loop is interlocked with the evening loop and morning loop. PRR5, PRR7, PRR9, and CCA1/LHY constitute the morning loop (Nakamichi et al., 2010; Salomé et al., 2010; Pokhilko et al., 2012), whereas PRR3, GI, ZTL (ZEITLUPE), and TOC1 comprise the evening loop (Kim et al., 2003; Mas et al., 2003b; Para et al., 2007; McClung and Gutiérrez, 2010). We found that LDL1 and CCA1 co-target to a subset of circadian genes, which are repressed by CCA1 in the morning. However, LDL1 also targets to the morning expressed circadian genes, which may not be repressed by CCA1 and LHY (Nagel et al., 2015; Kamioka et al., 2016; Hung et al., 2018). Although the binding of LDL1 on the LDL1/CCA1 co-targeted genes are reduced in the *cca1/lhy* mutant, their binding is not completely abolished (Hung et al., 2018). These results suggested that in addition to CCA1 and LHY, the LDL1/2-HDA6 complex may also functionally associate with other circadian clock genes. EC (Evening Complex) is also associated with regulation of the circadian genes, which is comprised of LUX (LUX ARRHYTHMO), ELF3 (EARLY FLOWERING3), and ELF4 (EARLY FLOWERING4) (Hazen et al., 2005; Nusinow et al., 2011). A previous study indicated that *Arabidopsis* HDACs are associated with PRR9 through direct interacting with TPL/TPR (TOPLESS/TOPLESS-RELATED) to regulate the expression of *CCA1* (Wang et al., 2013). Further research is required to investigate the functional correlation among LDL1/2-HDA6, PRR9, and EC.

The central loop of *Arabidopsis* circadian clock is consisted of the core clock components including CCA1, LHY, and TOC1 (Schaffer et al., 1998; Wang and Tobin, 1998; Alabadi et al., 2001; Gendron et al., 2012; Huang et al., 2012; Nagel et al., 2015). Although *CCA1* and *LHY* are low expressed at nightfall, they are highly induced at dawn (Schaffer et al., 1998; Wang and Tobin, 1998; Alabadi et al., 2001). Previously, we found that CCA1 interacts with LDL1 in the morning (Hung et al., 2018). The binding of LDL1 and HDA6 on promoter of *TOC1* is higher in the morning but decreased in the evening (Hung et al., 2018). Furthermore, *HDA6, LDL1*, and *LDL2* are constitutively expressed at different time periods. CCA1/LHY can therefore recruit the LDL1/2-HDA6 complex to suppress *TOC1* expression at dawn (Hung et al., 2018). In this study, we found that LDL1/2 and HDA6 also interact with TOC1 to regulate the expression of *CCA1* and *LHY*. In consistent with the fact that TOC1 is highly accumulated at nightfall (Alabadi et al., 2001), we found that the binding of LDL1 and HDA6 on the *CCA1* and *LHY* promoters is higher in the evening but decreased in the morning. Since TOC1 is a repressor of *CCA1* and *LHY*, the expression of *CCA1* and *LHY* is decreased with increased *TOC1* expression (Gendron et al., 2012; Huang et al., 2012). We found that histone acetylation and H3K4 methylation levels of *CCA1* and *LHY* are decreased in *TOC1-OE* plants. However, the H3Ac, H3K4me and expression levels of *CCA1* and *LHY* are significantly increased in *TOC1-OE/hda6/ldl1/2* compared to the *TOC1-OE* plants, indicating that the LDL1/2-HDA6 complex is functionally associated with the regulation of *CCA1* and *LHY* expression. Although the expression of *TOC1* is highly increased in *hda6/ldl1/2* compared to wild type, the expression of *CCA1* and *LHY* is not decreased. It is possible that in addition to LDL1/2-HDA6, other unknown proteins may also be involved in the regulation of *CCA1* and *LHY* expression.

**Figure 6:**
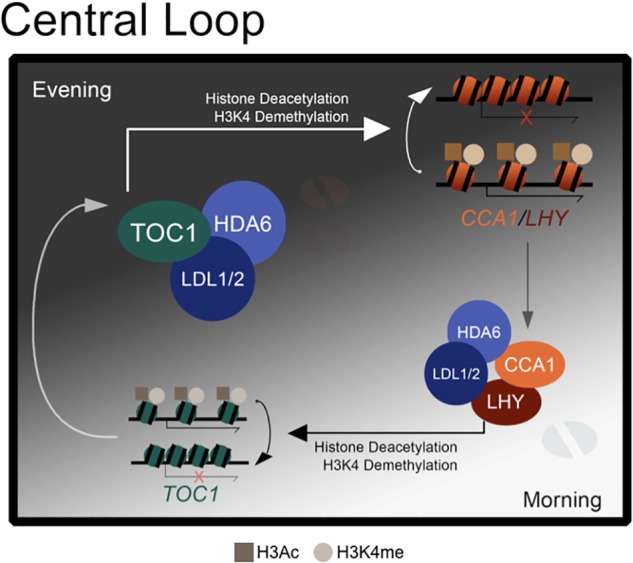
A model for LDL1/2 and HDA6 functions in the regulation of core circadian clock components. Both morning accumulated CCA1/LHY and evening accumulated TOC1 interact with the same histone modification complex containing LDL1/2 and HDA6. CCA1/LHY act as transcription repressors and recruit the histone modification complex to their target loci such as *TOC1* in the morning. Meanwhile, TOC1 also recruits the histone modification complex to its targets such as *CCA1* and *LHY* in the evening.

Collectively, we propose a model to demonstrate how the core circadian clock components are regulated by H3K4 demethylation and histone deacetylation (Figure 6). The histone modification complex containing LDL1/2 and HDA6 can interact with both morning accumulated CCA1/LHY (Hung et al., 2018) and evening accumulated TOC1. The transcription repressors CCA1 and LHY can recruit the LDL1/2-HDA6 complex to their target loci including *TOC1* in the morning (Hung et al., 2018). Furthermore, TOC1 can also recruit the histone modification complex to its targets such as *CCA1* and *LHY* in the evening.

## Data Availability

All datasets generated for this study are included in the manuscript and/or the Supplementary Files.

## Author Contributions

F-YH, KW, and YC designed the research. F-YH, F-FC, and J-HC performed the research. F-YH, CL, CC, and KW analyzed the data. F-YH and KW wrote the article.

## Conflict of Interest Statement

The authors declare that the research was conducted in the absence of any commercial or financial relationships that could be construed as a potential conflict of interest.

## Footnotes

1 https://www.psb.ugent.be/core-facilities/380-gateway-vectors

2 https://www.leica-microsystems.com/products/confocal-microscopes/p/leica-tcs-sp5/

3 https://www.lifetechnologies.com/
