# Supplementary material for: The LDL1/2-HDA6 Histone Modification Complex Interacts With TOC1 and Regulates the Core Circadian Clock Components in Arabidopsis
Source: Front Plant Sci. 2019 Feb 26;10:233. doi: 10.3389/fpls.2019.00233 (PMC6399392; doi:10.3389/fpls.2019.00233)
Supplement: Supplementary file 1 [file Table_1.pdf]

Table S1. Primers used in this research.

NAME	SEQUENCE	NOTE
CCA1_RT_F	TCTGTGTCTGACGAGGGTCGAATT	qRT-PCR
CCA1_RT_R	ACTTTGCGGCAATACCTCTCTGG	qRT-PCR
LHY_RT_F	ACGAAACAGGTAAGTGGCGACATT	qRT-PCR
LHY_RT_R	TGGGAACATCTTGAACCGCGTT	qRT-PCR
TOC1_RT_F	AGCTGCACCTAGCTTCAAGCACT	qRT-PCR
TOC1_RT_R	ATGATGTCGAGGCAAGACGAAGTC	qRT-PCR
UBQ10_F	TCCAGGACAAGGAGGTATTCCTCCG	qRT-PCR
UBQ10_R	CCACCAAAGTTTTACATGAAACGAA	qRT-PCR
CCA1_P_F	GTGTAGTGAACCGCACGAGA	ChIP
CCA1_P_R	CCGGGACTACCTGAAAGGTT	ChIP
CCA1_E_F	GAGGAGCTTAGTGATGGAGACA	ChIP
CCA1_E_R	TTCCTCAGTCCACCTTTCACG	ChIP
CCA1_U_F	CTTGTCCATAGTCTTCCTGTAACAT	ChIP
CCA1_U_R	GAGCCCCTTGAGTGAAGTTCT	ChIP
LHY_P_F	TTCTGGCTCGTAGAGAAGCAA	ChIP
LHY_P_R	GCCAGAAGCAATCTCAGCCAC	ChIP
LHY_E_F	TGTTTGGGGAGATCAAAGATGGT	ChIP
LHY_E_R	TGTATTAGTATCCATAACAGGACCG	ChIP
LHY_U_F	TCTTTGGTCATGTCAGGTTCTGT	ChIP
LHY_U_R	GACAAGAGACAAGACATGGGGT	ChIP
ACT2_F	CGTTTCGCTTTCCTTAGTGTTAGCT	ChIP
ACT2_R	AGCGAACGGATCTAGAGACTCACCTTG	ChIP
TUB2_F	ACAAACACAGAGAGGAGTGAGCA	ChIP
TUB2_R	ACGCATCTTCGGTTGGATGAGTGA	ChIP
