# Supplementary figures and images for: The LDL1/2-HDA6 Histone Modification Complex Interacts With TOC1 and Regulates the Core Circadian Clock Components in Arabidopsis
Source: Front Plant Sci. 2019 Feb 26;10:233. doi: 10.3389/fpls.2019.00233 (PMC6399392; doi:10.3389/fpls.2019.00233)

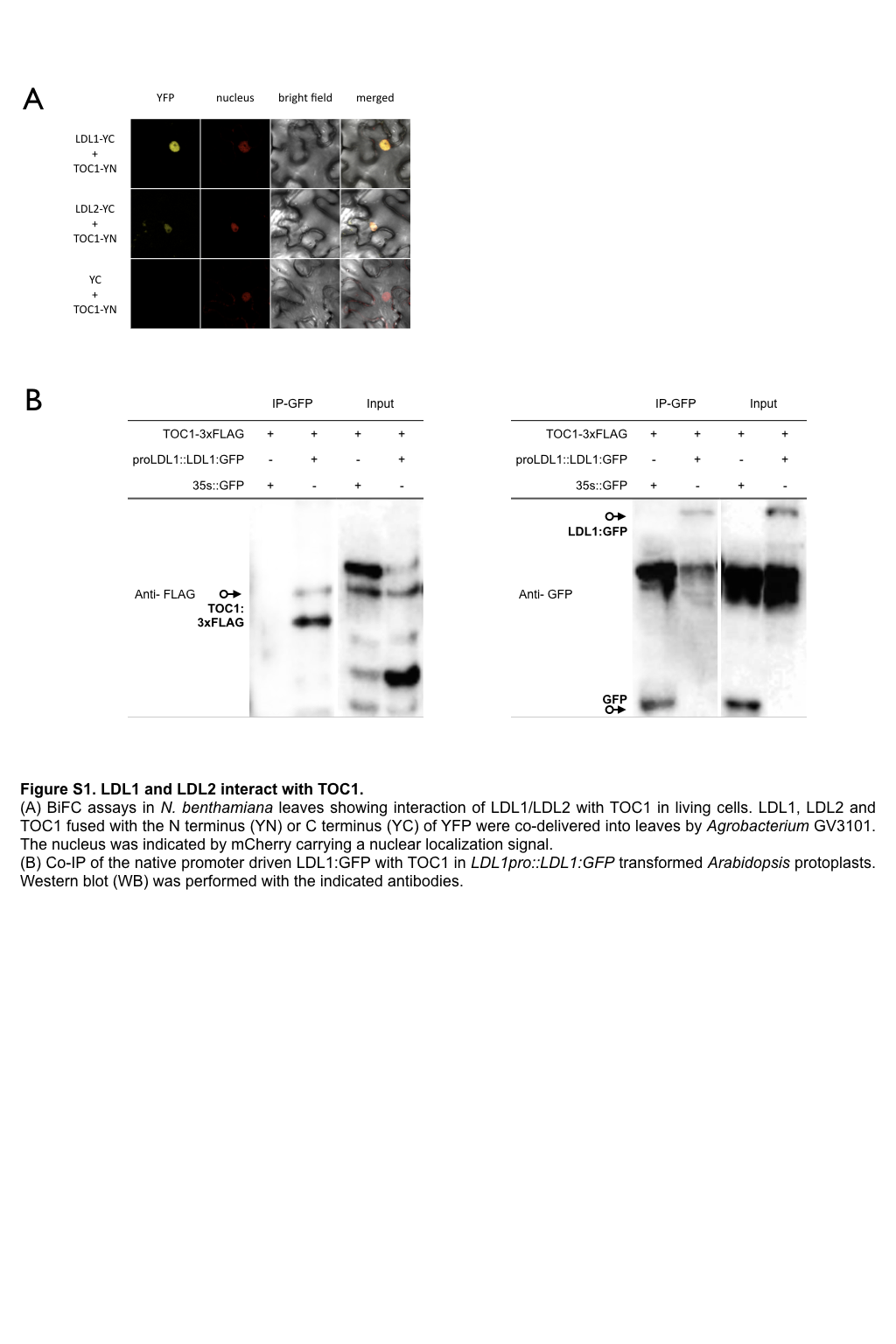

Supplement: Supplementary file 2 [file Image_1.TIFF]

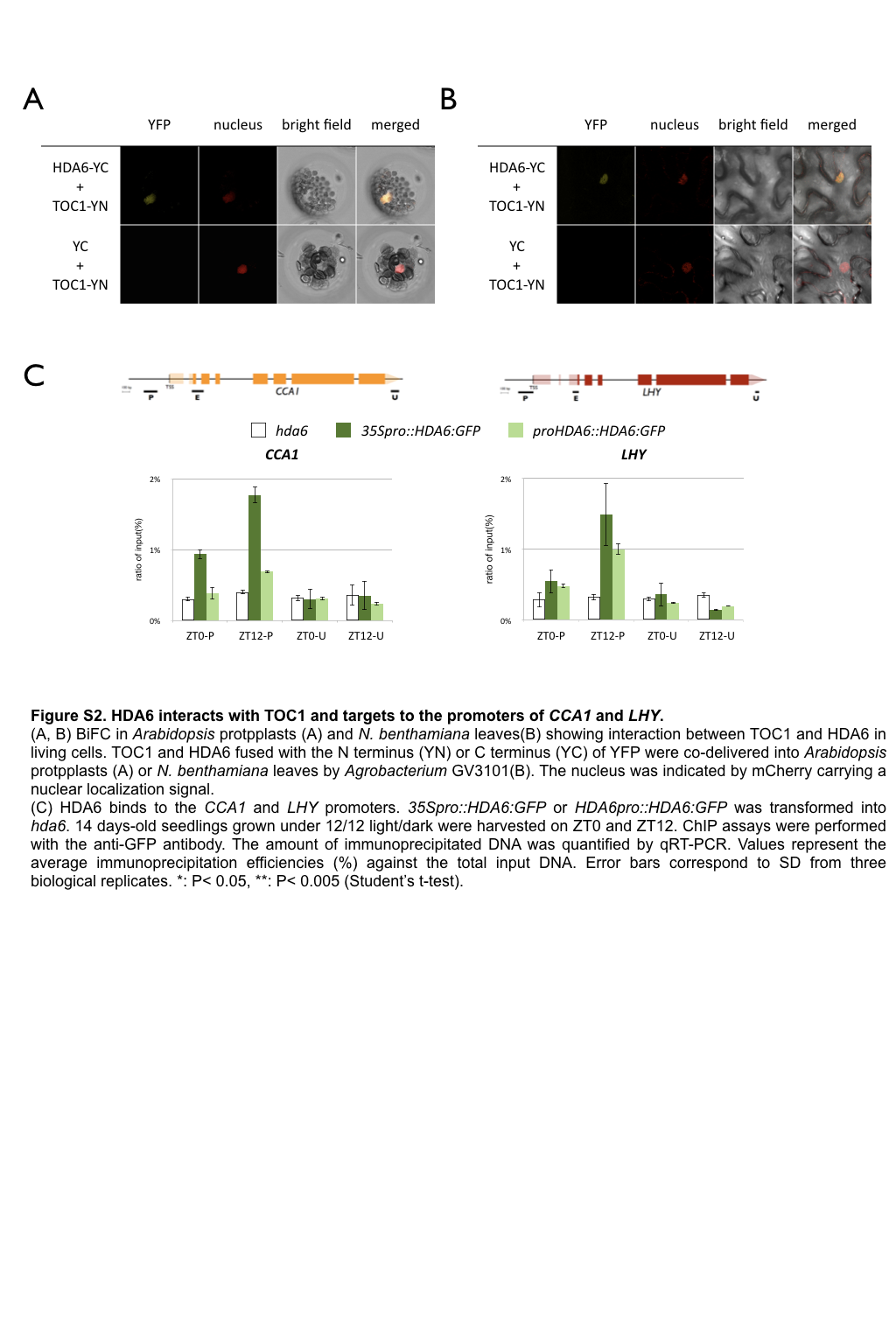

Supplement: Supplementary file 3 [file Image_2.PNG]

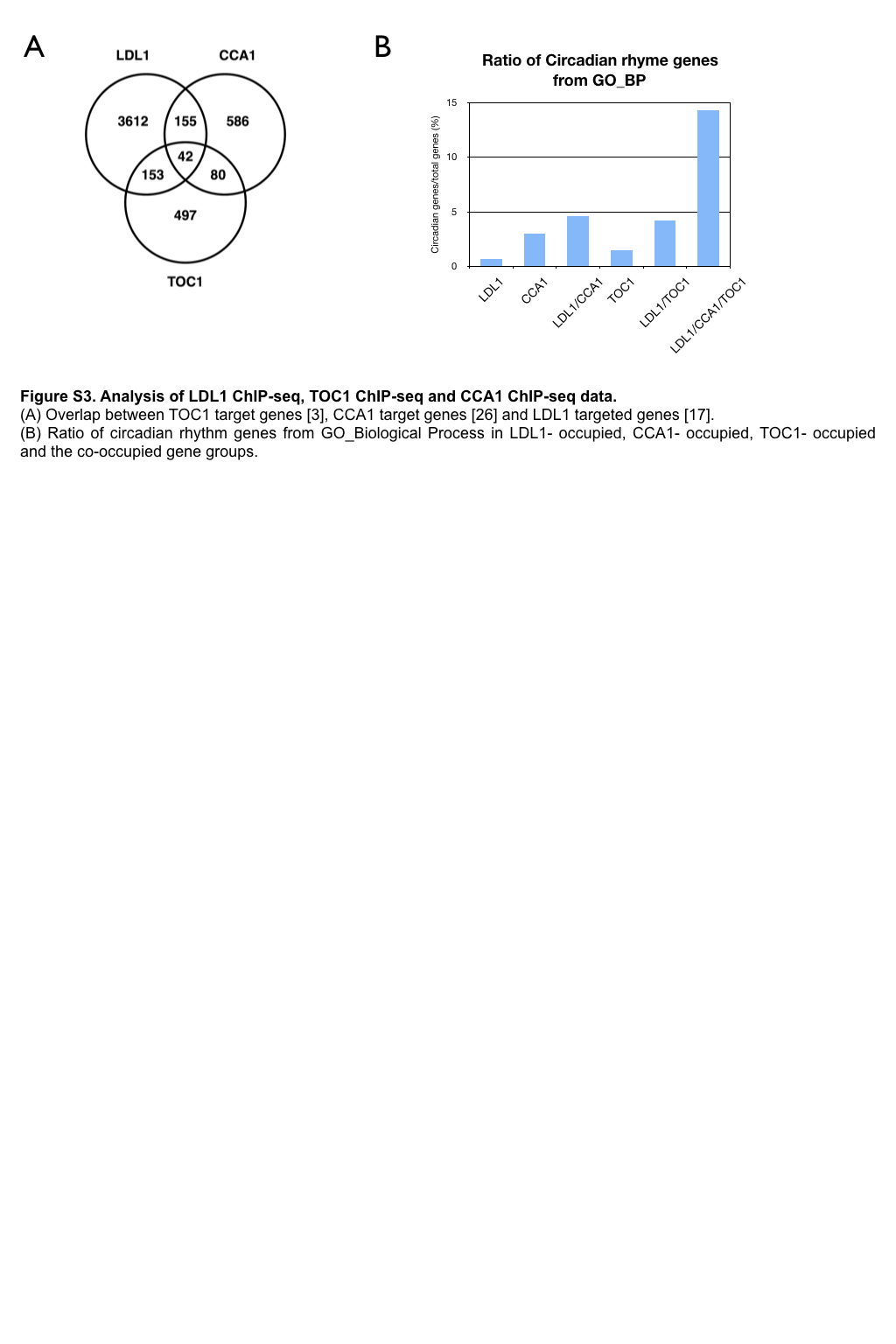

Supplement: Supplementary file 4 [file Image_3.PNG]

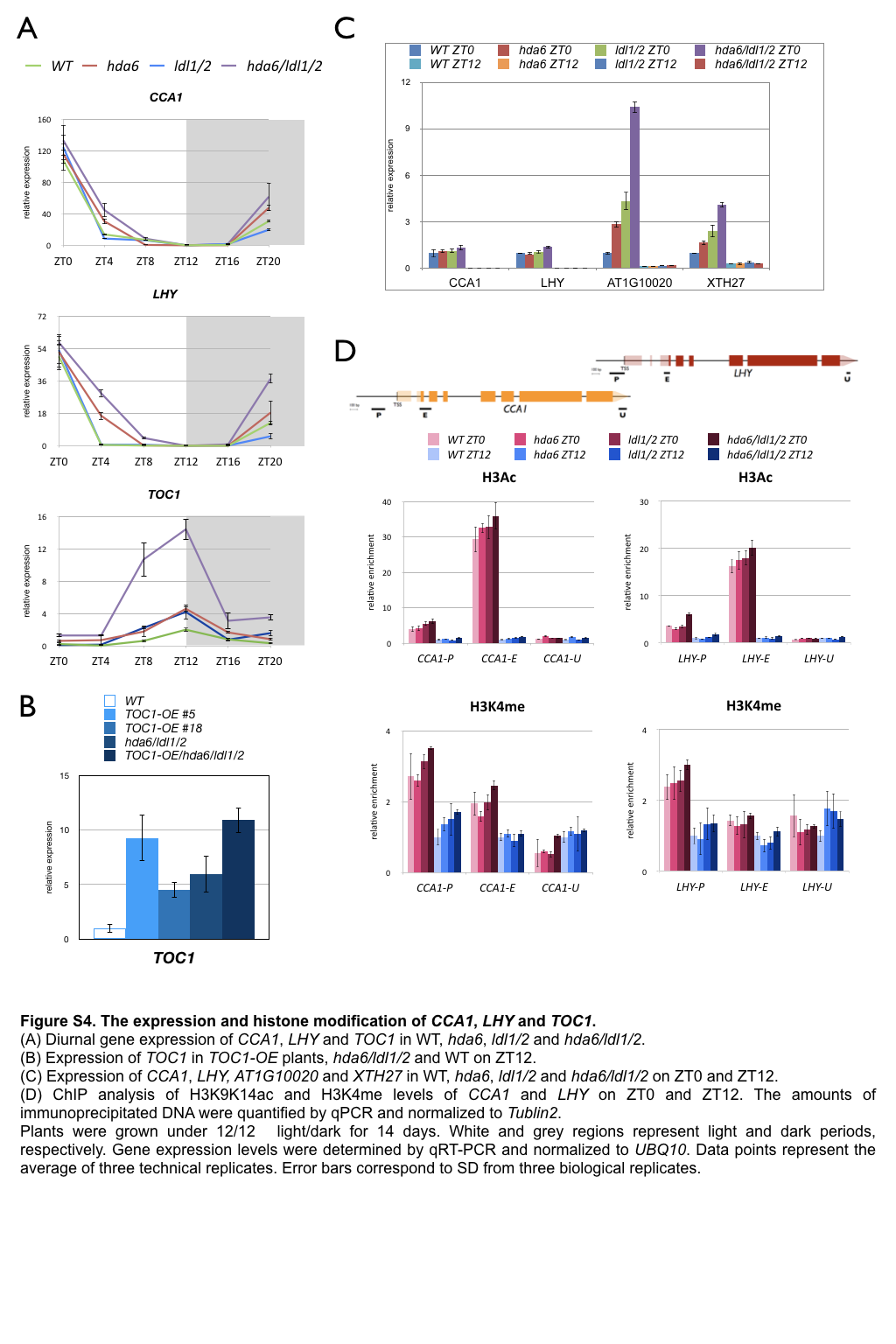

Supplement: Supplementary file 5 [file Image_4.TIFF]
